# Isolation and Characterization of the subcluster L2 Mycobacteriophage Underpass and its lysogen

**DOI:** 10.17912/micropub.biology.001915

**Published:** 2025-11-19

**Authors:** Kiley R. Toth, Alexa R. Rogers, Emma R. Wenzel, Farrah Ponnwitz, Maria-Lainie Galdo, Paul M. Lacour, Jose A. Torres Cruz, Milani Parikh, Inda B. Bard Hennessy, Nora K. Duval, Reilly A. Rosato, Rana M. Elshayet, Durva Joshi, Lauren Zile, Jessalyn F. Aquilino, John M. Braverman, C. Nicole Sunnen, Julia Y. Lee-Soety

**Affiliations:** 1 Department of Biology, Saint Joseph's University

## Abstract

We report here on mycobacteriophage Underpass, isolated from a soil sample collected at a construction site at Saint Joseph’s University in Philadelphia, Pennsylvania. Underpass is a siphovirus that infects *Mycobacterium smegmatis* mc ^2^ 155, and is able to establish lysogens. Within its 71,053 bp genome, we identified 134 predicted genes. Based on gene content similiarity to Actinobacteriophages, Underpass is a member of the L2 subcluster.

**Figure 1. Mycobacteriophage Underpass is a temperate siphovirus that forms stable lysogens:**
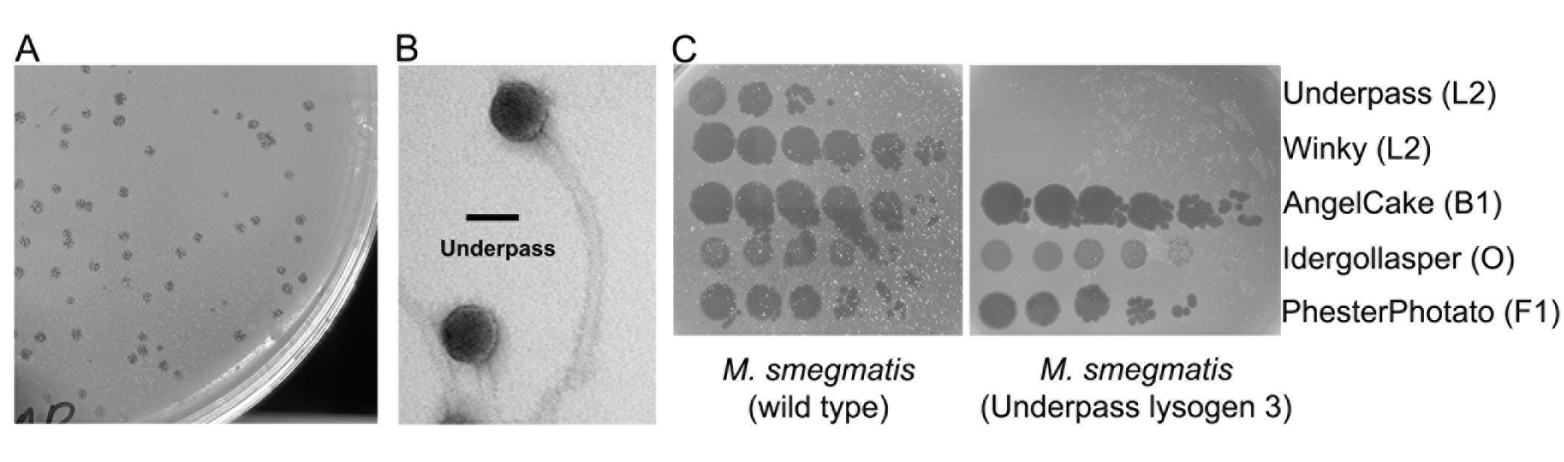
**A.** Underpass yielded lightly turbid plaques after a 48-hour incubation. **B.** Mycobacteriophage Underpass shows a siphovirus morphology with an icosahedral head measuring 48.3 nm ± 1.3 nm ( *N* = 6) in diameter and a long, flexible, noncontractile tail measuring 188.6 nm ± 34.9 nm ( *N* = 6) in length. High-titer lysate was stained with 1% uranyl acetate. Scale bar represents 50 nm. **C.** A representative Underpass lysogen is immune to secondary infection by Underpass and another subcluster L2 phage, Winky, but not by phages of other clusters.

## Description

Bacteriophages are viruses that infect, replicate in, and kill their bacterial host and therefore hold promise as a therapeutic for antimicrobial-resistant bacterial infections. Temperate bacteriophages, however, are able to integrate their genome into the host, transforming their host into lysogens that are resistant to phage superinfection and are therefore unsuitable for therapeutics. With the rise of antimicrobial-resistant bacteria, for example the pathogenic *Mycobacterium* spp., the isolation of novel phages and characterizing their lifecycle are important to advance the development of phages as therapeutics (Azimi et al., 2019; Dedrick et al., 2023; Hyman, 2019; Recchia et al., 2023; Zhou et al., 2023).

Here, we report on the temperate phage Underpass, which infects non-pathogenic *M. smegmatis* mc ^2^ 155 and was isolated from a soil sample collected on the Saint Joseph’s University Hawk Hill campus in Philadelphia, PA (39.99393 N, 75.24045 W), where a pedestrian underpass was being constructed. Phage Underpass was isolated by suspending the soil in 7H9 liquid medium, shaking at 37 °C for 1.5 hours, then filtering (0.2 μm pore size) the suspension. Thereafter, the filtrate was inoculated with *M. smegmatis* and incubated with shaking for two days at 37 °C. Following centrifugation, the filtered (0.2 μm pore size) supernatant was plated in top agar containing *M. smegmatis* to yield 1.8 ± 0.21 mm plaques ( *N* = 10) that were lightly turbid after a 48-hour incubation period (Fig 1A). Negative-stain transmission electron microscopy (1% uranyl acetate) revealed a siphovirus morphology ( Figure 1B ).

DNA was isolated from a lysate of Underpass by zinc-chloride precipitation (https://tinyurl.com/5fsarpd8), prepared for sequencing using the NEB Ultra II FS library kit, and sequenced on Illumina NextSeq 1000, yielding 1,323,933 single-end 100-base reads. Raw reads were trimmed with cutadapt 4.7 (using the option: nextseq-trim 30) and filtered with skewer 0.2.2 (using the options: -q 20 -Q 30 -n -l 50) prior to assembly (Gordon et al., 1998; Jiang et al., 2014; Martin, 2011; Wick et al., 2017). Assembled genome was checked for completeness using Newbler (v2.9) and Consed (v29) generating a single contig with 1809-fold coverage (Russell, 2018); the finalized genome was 71,053 bp, with a 10-base 3’ single-stranded genome termini and containing a G+C content of 58%. Underpass was assigned to the subcluster L2 based on clustering parameters of at least 35% shared gene content (GCS) to other phages within the Actinobacteriophage database, https://phagesdb.org (Pope et al., 2017).

Underpass’s genome was automatically annotated using Glimmer (Delcher et al., 1999) and Genemark (Besemer et al., 2001) through PECAAN (v20250130) (Rinehart CA, 2016). The auto-annotation was refined using Phamerator v602 using the Actino_draft database (Cresawn et al., 2011), Starterator v602 ( http://phages.wustl.edu/starterator ), Aragorn v1.2.41 and tRNAscanSE v2.0 (Chan and Lowe, 2019; Schattner et al., 2005), NCBI BLASTp v2.13.0+ searches against the Actinobacteriophage and NCBI non-redundant databases, and HHPred using the PDB_mmCIF70, Pfam- v.36, NCBI Conserved Domains databases (Altschul et al., 1990; Soding et al., 2005). All software was used with default parameters.

Underpass encodes 134 predicted genes. Putative functions were assigned to 58 protein-coding genes, and 12 tRNA genes were identified. Although Underpass largely conforms to the genome characteristics of 38 other fully sequenced phages assigned to the L2 subcluster, to date, in terms of G+C content and number of tRNAs, it has one of the smallest genomes, lacking an ~ 5 kb region present in the right arm of larger L2 phages, which are up to 76,366 bp. This region contains 9 genes, one of which encodes a Ro-like RNA binding protein, which is likely involved but not required in the post-transcriptional regulation of phage development (Altuvia et al., 2018; Uzan and Miller, 2010).

Underpass encodes for a tyrosine integrase (gp38) and an immunity repressor (gp40), and is thus predicted to be temperate; this is supported by experimental evidence of lysogen formation. Briefly, 3 μl of an Underpass lysate was spotted on a top agar lawn with *M. smegmatis.* Following incubation at 37˚C for 3 days, bacteria that formed a mesa within the spot were collected, streaked onto an agar plate to isolate candidate lysogen colonies, which were then purified by streaking 4 additional times. Liquid cultures were then prepared by inoculating 7H9 liquid medium with individual candidate lysogen colonies and incubating the mixture with shaking for two days at 37˚C. Each culture was then spun and the supernatant spotted on a top agar lawn with *M. smegmatis.* The resulting spot clearings on the lawn of *M. smegmatis* was indicative of spontaneous induction and phage release that is typical of lysogens (Galdo et al., 2025). The same cultures were also immune to secondary infection, or superinfection, by Underpass and Winky (another L2 phage) but not by AngelCake (cluster B1), Idergollasper (O), and Phesterphotato (F1) ( Figure 1C ).

**Nucleotide sequence accession numbers**

Underpass is available at GenBank with Accession No. PV940982 and Sequence Read Archive (SRA) No. SRX29486061.
